# Sevoflurane and nephrogenic diabetes insipidus on the rise: copeptin to the rescue?

**DOI:** 10.1186/s13054-019-2594-3

**Published:** 2019-09-05

**Authors:** Patrick M. Honore, David De Bels, Leonel Barreto Gutierrez, Sebastien Redant, Andrea Gallerani, Willem Boer

**Affiliations:** 1 ICU Department, Centre Hospitalier Universitaire Brugmann, Place Van Gehuchtenplein, 4, Brussels, Belgium; 2 Department of Anesthesiology, Intensive Care Medicine, Emergency Medicine & Pain Medicine, Ziekenhuis Oost-Limburg, Genk, Belgium

With interest, we read the recent paper by Nigro et al. on the differential diagnosis of hypernatremia in intensive care unit (ICU) [1]. One of their main findings was an evidently higher copeptin levels in nephrogenic diabetes insipidus (NDI), compared to central diabetes insipidus (CDI). When choosing a cutoff value of < 4.4 pmol/L, copeptin levels had a sensitivity of 100% and a specificity of 99% for diagnosing CDI, whereas the values for NDI were approximately 18 times higher [1]. These findings confirmed the results of an earlier study by Timper et al. [2]. While the authors claim that the incidence of NDI remains very low, this may change with the increasing use of sevoflurane in ICU, which appears to be effective in reducing wake-up and extubation times and was recently described for sedation in acute respiratory distress syndrome [3]. In a recent retrospective study, of the 35 patients receiving sevoflurane, seven patients presented with NDI during their ICU stay (i.e., 20%) [3]. The main finding of this study was that sevoflurane sedation in ICU was associated with NDI in relation to concentration and duration of exposure. All patients with NDI were sedated with mean end-tidal concentration of sevoflurane > 1.0 vol% and for more than 72 h. Cabibel et al. also described the occurrence of three cases of NDI after 4 days or more of 1 vol% end-tidal sevoflurane administration [4]. It is thought that sevoflurane temporarily reduces urinary concentration capacity mechanisms allowing aquaporin 2 expression on cellular membranes [5]. Alternatively inorganic fluoride produced with hexafluoroisopropanolol during sevoflurane metabolism by cytochrome P450 may be toxic [4]. The differential diagnosis between NDI and CDI can be problematic as both groups may present with neurological problems [1]. Only a trial dose of desmopressin could differentiate as most but not all with NDI will remain unresponsive [3, 4]. Copeptin may resolve the diagnosis dilemma [1] allowing institution of appropriate treatment, either by removal of the cause (sevoflurane) or the treatment of the causality (brain edema, cerebral bleeding, etc.). In summary, as long as sevoflurane use increases and in the knowledge that 20% of these cases develop NDI, copeptin is a valuable tool permitting a rapid and definitive differential diagnosis between CDI and NDI.

## Data Availability

Not applicable.

## Abbreviations

ICU: Intensive care unit
NDI: Nephrogenic diabetes insipidus
CDI: Central diabetes insipidus
